# Is there a place for routine use of cemented acetabular fixation in primary total hip arthroplasty for osteoarthritis? Equivalence demonstrated in an analysis of 96,574 cases from the Australian Orthopaedic Association National Joint Replacement Registry

**DOI:** 10.1177/11207000261430706

**Published:** 2026-04-10

**Authors:** Richard J Hanly, Sarah L Whitehouse, Carl Holder, Richard de Steiger, Christopher J Wall, A John Timperley, Ross W Crawford, Dirk van Bavel

**Affiliations:** 1 Royal Devon University Healthcare NHS Foundation Trust, Exeter Hip Unit, Princess Elizabeth Orthopaedic Centre, Exeter, UK; 2 Lower Limb Clinic Brisbane, Brisbane, QLD, Australia; 3 Orthopaedic Research Unit, Queensland University of Technology, Chermside, QLD, Australia; 4 South Australian Health and Medical Research Institute (SAHMRI), Adelaide, SA, Australia; 5 Australian Orthopaedic Association National Joint Replacement Registry, Adelaide, SA, Australia; 6 University of Exeter, Exeter, UK; 7 Epworth Healthcare, Melbourne, VIC, Australia

**Keywords:** Cemented cup, cemented hip, registry, survival

## Abstract

**Introduction/Aim:**

This study utilises Australian Orthopaedic Association National Joint Replacement Registry (AOANJRR) data to compare survivorship of total hip arthroplasty (THA) with cemented and cementless acetabular fixation. To minimise confounders, only cases using cemented polished tapered stems with metal heads and highly cross-linked polyethylene (XLPE) liners for primary diagnosis of osteoarthritis were included.

**Methods:**

AOANJRR data were analysed from 01 September 1999 to 31 December 2022. The study population included all primary conventional THA performed for osteoarthritis using a cemented polished tapered stem with a bearing surface of metal head articulating with XLPE. These procedures were divided into two groups: procedures with either cemented or cementless acetabular fixation. The analyses were undertaken using Kaplan-Meier estimates of survivorship and hazard ratios (HR) from Cox proportional hazards models. Results were stratified by age and 3 surgeon volume groups: ⩽10, 10–25, and >25 procedures per surgeon per year.

**Results:**

There were 96,574 THAs performed for osteoarthritis using a cemented polished tapered stem included. Of these, 5,926 used cemented and 90,648 used cementless acetabular fixation. There was no difference in the rate of revision when cemented acetabular were compared to cementless acetabular procedures (HR 1.01; 95% CI, 0.86–1.18; *p* = 0.916). The most common reasons for revision surgery were similar for both fixation methods and included periprosthetic fracture, dislocation/instability and infection. There were no differences in the rate of revision when procedures were stratified by age or surgeon volume.

**Conclusions:**

For patients undergoing a primary THA for osteoarthritis there is no difference in the rate of revision between cemented or cementless acetabular fixation when utilising a cemented polished stem with modern bearing surfaces. The authors advocate the use of cemented acetabular fixation as a viable alternative and emphasise the need for continued teaching of the skills required to ensure the technique is not lost for future generations of orthopaedic surgeons.

## Background

Total hip arthroplasty (THA) advancements in materials, technique, and technology have shown demonstrable gains evidenced by reduced rates of revision in registries worldwide. Perceived limitations to survivorship with cemented fixation of the acetabular component led to the introduction of cementless technology in the 1980s. A myriad of basic science research has refuted the theories of “cement disease”^1^ as the prime cause of periprosthetic osteolysis, and clinical studies have demonstrated cemented fixation as having superior survivorship when examining beyond the first postoperative decade.^2^ The Australian Orthopaedic Association National Joint Replacement Registry (AOANJRR) reflects the global trend toward predominance of cementless acetabular fixation, reporting that only 1.9% of acetabular fixation in 2022 was with cement, down from 2.9% in 2019 and 14.1% in 2002.^3^ Similarly, the National Joint Registry (NJR) report that the use of fully cemented hips in the UK has decreased from 53.2% in 2004 to 17.1% in 2023.^4^ The paradoxical decline in use of cemented acetabular components despite proven survivorship is central to this analysis.^4^

National joint registries have tremendous utility for surveillance and identification of aberrant prostheses with early failure rates.^5^ They have robust external validity and are useful in guiding evidence-based practice.^6^ Surgeon volume, prosthesis choice, and modified polyethylene have previously been identified as independent variables affecting revision risk in the AOANJRR, and reflected in international registries.^7,8^ Temporal relations are also legitimate confounders in the comparison of survivorship of acetabular component fixation. Specifically relating to acetabular components is the effect, and timing, of the introduction of highly cross-linked polyethylene (XLPE).^9^ The AOANJRR classifies XLPE as ultra-high-molecular-weight polyethylene that has been irradiated by high dose (⩾50 kGy) gamma or electron beam radiation.^3^ XLPE has demonstrable decreases in *in vivo* wear and resultant decreases in revision for aseptic loosening.^10,11^

As an example of the disparity in the introduction of XLPE for cementless and cemented prostheses, there was a 5-year lag in the introduction of Rimfit X3 cemented cups (Stryker, Mahwah, NJ, USA), following the release of Trident X3 liners (Stryker, Mahwah, NJ) within a single brand portfolio,^9^ and Crossfire XLPE in the Trident cup prior to this from their introduction in 1998. This polyethylene bias has been recognised, and the reported survivorship advantages of hybrid THA over cemented THA at all time points and age stratifications from the 2016 AOANJRR Annual Report were nullified with the exclusion of non-modified polyethylene in the 2017 iteration.^7,12^ Registries globally are disparate in making this distinction, leading to continued potential for polyethylene bias in reporting. In 2018, the AOANJRR reported that XLPE had a significantly lower rate of revision compared to non-XLPE.^13^ The cumulative percent revision (CPR) for THA performed with XLPE was 6.2% at 16 years compared to 11.1% for non XLPE.^13,14^ The 2023 AOANJRR Annual Report demonstrates a continued survivorship divergence with 20 year CPR of 6.9% for XLPE and 17.2% for non-XLPE.^3^

Utilising data from the AOANJRR, the aim of this paper was to compare the survivorship of cemented and cementless acetabular fixation using XLPE and a cemented polished tapered stem for patients undergoing THA for a primary diagnosis of osteoarthritis.

## Methods

We conducted a comparative cohort study using data from the AOANJRR from 01 September 1999 until 31 December 2022. The AOANJRR commenced data collection on 01 September 1999, achieving complete national implementation by mid-2002. Since then, it has collected data on 99% of THA and total knee arthroplasties (TKA) performed in Australia.^3^

These data are externally validated against patient-level data provided by all Australian state and territory health departments. A sequential, multilevel matching process is used to identify any missing data which are subsequently obtained by follow-up with the relevant hospital. Each month, in addition to internal validation and data quality checks, all primary procedures are linked to any subsequent revision involving the same patient, joint and side. A revision is defined as the removal, replacement, or addition of any device component. Major revision involves the femoral stem and/or the acetabular cup/shell. Minor revision involves exchange of the femoral head and/or acetabular liner.^15^ Data are also matched bi-annually to the Australian National Death Index data to identify patients who have died.

In 2020, the AOANJRR commenced collection of patient-reported outcome measures (PROMs) including EuroQol 5-dimension health questionnaire (EQ-5D), EuroQol visual analogue scale (EQ-VAS) and Oxford Hip Score (OHS), in addition to several questions pertaining to pain, expectations, and satisfaction. PROMs are collected preoperatively and at 6 months postoperatively.^16^ Available PROMs for this cohort have been included in this analysis.

The study population included all primary conventional THA undertaken for osteoarthritis using a cemented polished tapered stem with a bearing surface consisting of a metal head articulating with XLPE. Ceramic heads were excluded from this analysis. These procedures were divided into 2 groups: procedures with cemented acetabular fixation, and procedures with cementless acetabular fixation. As the use of cemented cups with cementless stems is rare, all cementless stems have been excluded. Matte finished stems have been reported as having a significantly higher revision rate and have been excluded from this study.^17^ There were 495 THA procedures which were excluded: 483 with cementless acetabular components that had been cemented, 10 cemented cups inserted without cement, and 2 reverse hybrid (femur cementless) procedures. To increase the internal validity of this analysis, results were further stratified in separate analyses by age and 3 surgeon volume groups: ⩽10, 10–25, and >25 procedures per surgeon per year.

### Statistical analysis

Kaplan-Meier estimates of survivorship were used to report the time to revision, with censoring at the time of death and closure of the dataset at the end of December 2022. The unadjusted cumulative percent revision (CPR), with 95% confidence intervals (CI), was calculated using unadjusted point wise Greenwood estimates. Age and sex adjusted hazard ratios (HR) were calculated from Cox proportional hazard models to compare the rate of revision between groups. The assumption of proportional hazards was checked analytically for each model. If the interaction between the predictor and the log of time was statistically significant in the standard Cox model, then a time varying model was estimated. Time points were selected based on the greatest change in hazard, weighted by a function of events. Time points were iteratively chosen until the assumption of proportionality was met and HRs were calculated for each selected time-period. For the current study, if no time-period was specified, the HR was calculated over the entire follow-up period. All tests were 2-tailed at 5% levels of significance. Statistical analysis was performed using SAS software version 9.4 (SAS Institute Inc., Cary, NC, USA).

## Results

There were 96,574 THA procedures performed for osteoarthritis using a cemented polished tapered stem included in this analysis. Of these, 5,926 used cemented acetabular fixation and 90,648 used cementless acetabular fixation. The combinations of femoral and acetabular prostheses stratified by acetabular fixation are presented in Tables 1 and 2.

**Table 1.** Cemented primary total conventional hip arthroplasty by prosthesis combination (primary diagnosis osteoarthritis).

Prosthesis Combination	Number
Exeter V40/Exeter X3 Rimfit	3917
C-Stem AMT/Marathon	447
CPCS/Reflection (Cup)	434
Short Exeter V40/Exeter X3 Rimfit	372
CPT/ZCA	220
MS 30/ZCA	125
Other (51)	411
**TOTAL**	**5926**

Note: Only combinations with >100 procedures have been listed.

**Table 2.** Hybrid (femur cemented) primary total conventional hip arthroplasty by femoral and acetabular component (primary diagnosis osteoarthritis).

Prosthesis Combination	Number
Exeter V40/Trident (Shell)	51214
CPT/Trilogy	6516
C-Stem AMT/PINNACLE	3500
CPCS/R3	2883
Exeter V40/Trident/Tritanium (Shell)	2838
Short Exeter V40/Trident (Shell)	2302
CPT/Continuum	2112
Exeter V40/R3	1756
CPT/Trabecular Metal (Shell)	1576
Quadra-C/Mpact	1562
CPCS/Reflection (Shell)	1522
CPT/Allofit	1436
Exeter V40/PINNACLE	1018
MS 30/Allofit	974
Exeter V40/Vitalock	783
C-Stem/PINNACLE	716
Quadra-C/Versafitcup CC	633
Exeter V40/Mallory-Head	422
MS 30/Fitmore	411
C-Stem/Duraloc	392
MS 30/Continuum	359
Evolve/Logical G	357
Exeter V40/Trilogy	354
E2/C2	342
VerSys Heritage/Trilogy	322
Exeter V40/Trabecular Metal (Shell)	320
Absolut/Acetabular Shell (Global)	311
E2/Logical G	267
X-Acta/Versafitcup CC	259
Exeter V40/Trident II (Shell)	224
C-Stem AMT/Trilogy	219
X-Acta/Mpact	199
Exeter V40/Restoration	174
Exeter/Trident (Shell)	144
CPT/Fitmore	141
Exeter V40/Fixa	139
Short Exeter V40/Trident/Tritanium (Shell)	135
MS 30/PINNACLE	133
Other (136)	1683
**TOTAL**	**90648**

Note: Only combinations with >100 procedures have been listed.

The mean age of patients was 73.3 years (± 9.2 years) (75.3 years for cemented and 73.2 years for cementless) with a predominance of female patients in both groups (67.6% and 61.3%, respectively). The maximum follow-up was 19.4 years for the cemented group and 22.7 years for the cementless group (Table 3).

**Table 3.** Summary of primary total conventional hip arthroplasty by fixation (primary diagnosis osteoarthritis).

Variable		Cemented	Hybrid (Femur Cemented)	TOTAL
Follow-up years				
	Mean ± SD	5.9 ± 3.5	7.1 ± 4.7	7 ± 4.7
	Median (IQR)	5.8 (3, 8.6)	6.4 (3.3, 10.1)	6.4 (3.3, 10)
	Minimum	0	0	0
	Maximum	19.4	22.7	22.7
Age				
	Mean ± SD	75.3 ± 9.6	73.2 ± 9.2	73.3 ± 9.2
	Median (IQR)	76 (70, 82)	74 (68, 80)	74 (68, 80)
Age Group				
	<55	183 (3.1%)	3,119 (3.4%)	3,302 (3.4%)
	55–64	512 (8.6%)	11,913 (13.1%)	12,425 (12.9%)
	65–74	1,780 (30%)	32,286 (35.6%)	34,066 (35.3%)
	⩾75	3,451 (58.2%)	43,330 (47.8%)	46,781 (48.4%)
Sex				
	Male	1,922 (32.4%)	35,073 (38.7%)	36,995 (38.3%)
	Female	4,004 (67.6%)	55,575 (61.3%)	59,579 (61.7%)
Femoral head size				
	<28 mm	98 (1.7%)	2,146 (2.4%)	2,244 (2.3%)
	28 mm	1,000 (16.9%)	21,887 (24.1%)	22,887 (23.7%)
	32 mm	4,117 (69.5%)	41,443 (45.7%)	45,560 (47.2%)
	>32 mm	711 (12.0%)	25,172 (27.8%)	25,883 (26.8%)
ASA score^a^				
	1	108 (2.4%)	2,114 (4%)	2,222 (3.9%)
	2	2,070 (46.3%)	26,003 (49.5%)	28,073 (49.3%)
	3	2,151 (48.1%)	23,221 (44.2%)	25,372 (44.5%)
	4	139 (3.1%)	1,151 (2.2%)	1,290 (2.3%)
	5		3 (0%)	3 (0%)
BMI^b^				
	Underweight (<18.50)	38 (1.2%)	409 (1%)	447 (1%)
	Normal (18.50-24.99)	871 (26.5%)	8,933 (21.5%)	9,804 (21.8%)
	Pre Obese (25.00-29.99)	1,237 (37.6%)	15,060 (36.2%)	16,297 (36.3%)
	Obese Class 1 (30.00-34.99)	732 (22.2%)	10,555 (25.4%)	11,287 (25.1%)
	Obese Class 2 (35.00-39.99)	289 (8.8%)	4,496 (10.8%)	4,785 (10.7%)
	Obese Class 3 (⩾40.00)	126 (3.8%)	2,162 (5.2%)	2,288 (5.1%)
TOTAL		5,926	90,648	96,574

SD, standard deviation; IQR, interquartile range; ASA, American Society of Anesthesiologists; BMI, body mass index (kg/m^2^).

a Excludes 39,614 procedures with unknown ASA score.

b Excludes 51,666 procedures with unknown BMI.

**Figure 1.**
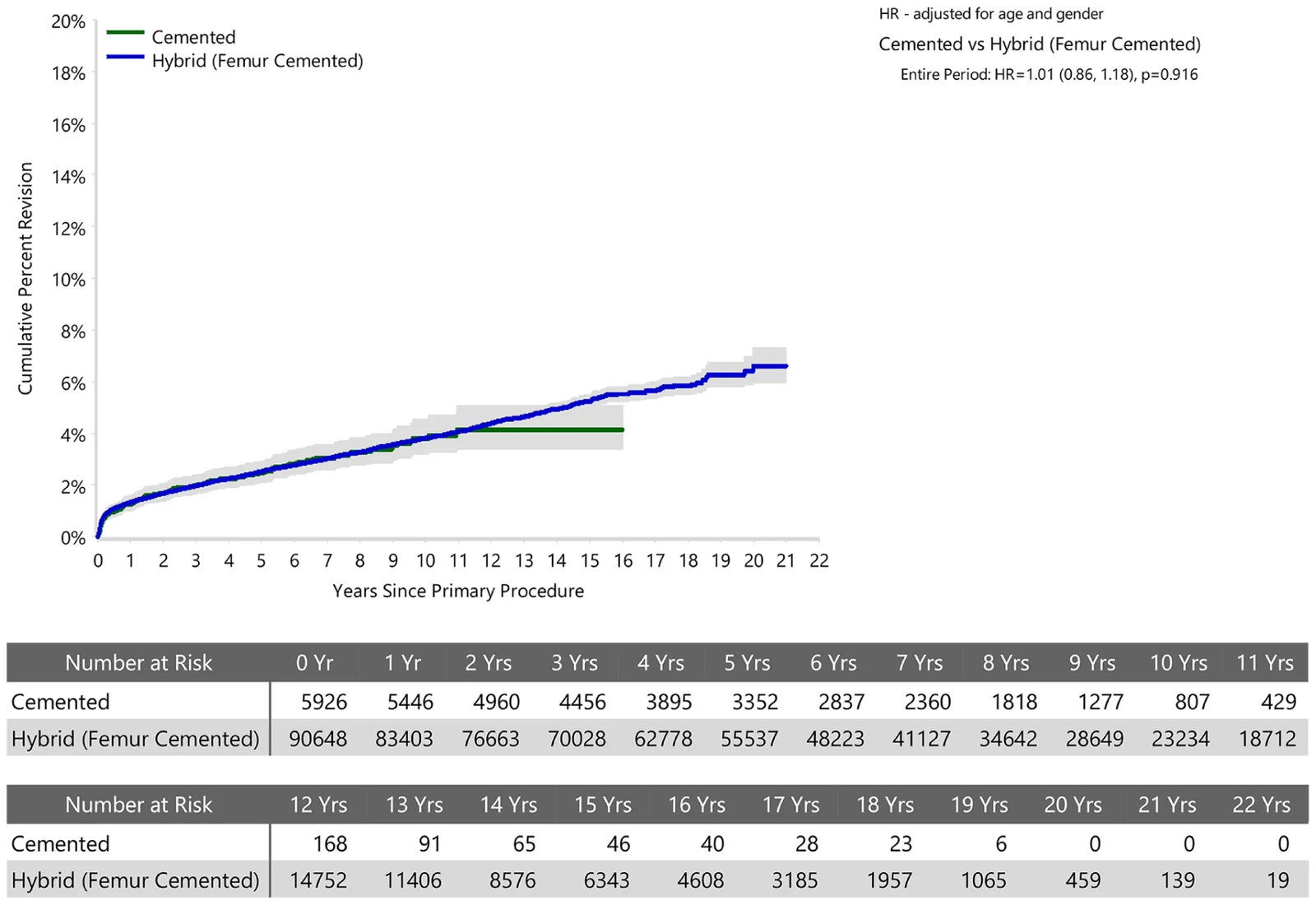
Cumulative Percent Revision of Primary Total Conventional Hip Arthroplasty by Fixation (Primary Diagnosis Osteoarthritis).

The CPR at 16 years was 4.1% (95% CI 3.4, 5.1) for cemented acetabular procedures and 5.5% (95% CI 5.2, 5.8) for cementless acetabular procedures (Figure 1). There was no significant difference in the rate of revision when comparing acetabular fixation method (HR 1.01; 95% CI 0.86, 1.18; *p* = 0.916) (Figure 1).

The most common reasons for revision for cemented acetabular procedures were fracture (0.8%, *n* = 49/161), infection (0.8%, *n* = 46/161), and prosthesis dislocation (0.6%, 36/161). For cementless acetabular components, prosthesis dislocation/instability (0.8%, *n* = 755/2787), infection (0.8%, 751/2787), and fracture (0.8%, 711/2787) were the most common revision diagnoses (Table 4). The type of revision for the most common reasons for revision stratified by acetabular fixation is presented (Tables 5–8) sub classified by componentry revised.

**Table 4.** Revision diagnosis of primary total conventional hip arthroplasty by fixation (primary diagnosis osteoarthritis) (follow-up limited to 19.4 years).

	Cemented			Hybrid (femur cemented)		
Revision diagnosis	Number	% Primaries revised	% Revisions	Number	% Primaries revised	% Revisions
Prosthesis Dislocation/Instability	36	0.6	22.4	755	0.8	27.1
Infection	46	0.8	28.6	751	0.8	26.9
Fracture	49	0.8	30.4	711	0.8	25.5
Loosening	26	0.4	16.1	351	0.4	12.6
Implant breakage stem	1	0.0	0.6	41	0.0	1.5
Pain	2	0.0	1.2	31	0.0	1.1
Malposition				28	0.0	1.0
Leg-length discrepancy				25	0.0	0.9
Lysis				17	0.0	0.6
Incorrect sizing				10	0.0	0.4
Wear acetabular insert				8	0.0	0.3
Metal related pathology				7	0.0	0.3
Implant breakage acetabular insert				6	0.0	0.2
Implant breakage acetabular				3	0.0	0.1
Heterotopic bone				2	0.0	0.1
Tumour				2	0.0	0.1
Wear acetabulum				1	0.0	0.0
Wear head				1	0.0	0.0
Other	1	0.0	0.6	37	0.0	1.3
***n* Revision**	**161**	**2.7**	**100.0**	**2787**	**3.1**	**100.0**
***n* Primary**	**5926**			**90648**		

Note: This table is restricted to revisions within 19.4 years for all groups to allow a time-matched comparison of revisions.

**Table 5.** Type of revision of primary total conventional hip arthroplasty by fixation (primary diagnosis osteoarthritis, revision for revision - prosthesis dislocation/instability).

	Cemented		Hybrid (Femur Cemented)		TOTAL	
Type of revision	*n*	Col%	*n*	Col%	*n*	Col%
Acetabular Component	23	63.9	209	27.7	232	29.3
THA (femoral/acetabular)	7	19.4	57	7.5	64	8.1
Femoral component	2	5.6	56	7.4	58	7.3
Minor revision	4	11.1	428	56.7	432	54.6
Other	0	0.0	5	0.7	5	0.6
**TOTAL**	**36**	**100.0**	**755**	**100.0**	**791**	**100.0**

Note: “Other” includes removal of prostheses, reinsertion of components or insertion of minor components.

This table is restricted to revisions within 19.4 years for all groups to allow a time-matched comparison of revisions.

**Table 6.** Type of revision of primary total conventional hip arthroplasty by fixation (primary diagnosis osteoarthritis, revision for revision - infection).

	Cemented		Hybrid (femur cemented)		TOTAL	
Type of revision	*n*	Col%	*n*	Col%	*n*	Col%
Minor revision	15	32.6	425	56.6	440	55.2
Major revision	31	67.4	326	43.4	357	44.8
**TOTAL**	**46**	**100.0**	**751**	**100.0**	**797**	**100.0**

Note: This table is restricted to revisions within 19.4 years for all groups to allow a time-matched comparison of revisions.

Major revision involves the femoral stem and/or the acetabular cup/shell. Minor revision involves exchange of the femoral head and/or acetabular liner.

**Table 7.** Type of revision of primary total conventional hip arthroplasty by fixation (primary diagnosis osteoarthritis, revision for revision - fracture).

	Cemented		Hybrid (femur cemented)		TOTAL	
Type of revision	*n*	Col%	*n*	Col%	*n*	Col%
Femoral component	39	79.6	579	81.4	618	81.3
THA (femoral/acetabular)	7	14.3	30	4.2	37	4.9
Acetabular component	0	0.0	33	4.6	33	4.3
Other (includes minor revision)	3	6.1	69	9.7	72	9.5
**TOTAL**	49	100.0	711	100.0	760	100.0

Note: This table is restricted to revisions within 19.4 years for all groups to allow a time-matched comparison of revisions.

**Table 8.** Type of revision of primary total conventional hip arthroplasty by fixation (primary diagnosis osteoarthritis, revision for revision - loosening).

	Cemented		Hybrid (femur cemented)		TOTAL	
Type of revision	*n*	Col%	*n*	Col%	*n*	Col%
Femoral component	2	7.7	135	38.5	137	36.3
Acetabular component	13	50.0	121	34.5	134	35.5
THR (femoral/acetabular)	11	42.3	52	14.8	63	16.7
Other (includes minor revision)	0	0.0	43	12.3	43	11.4
**TOTAL**	26	100.0	351	100.0	377	100.0

Note: This table is restricted to revisions within 19.4 years for all groups to allow a time-matched comparison of revisions.

**Figure 2.**
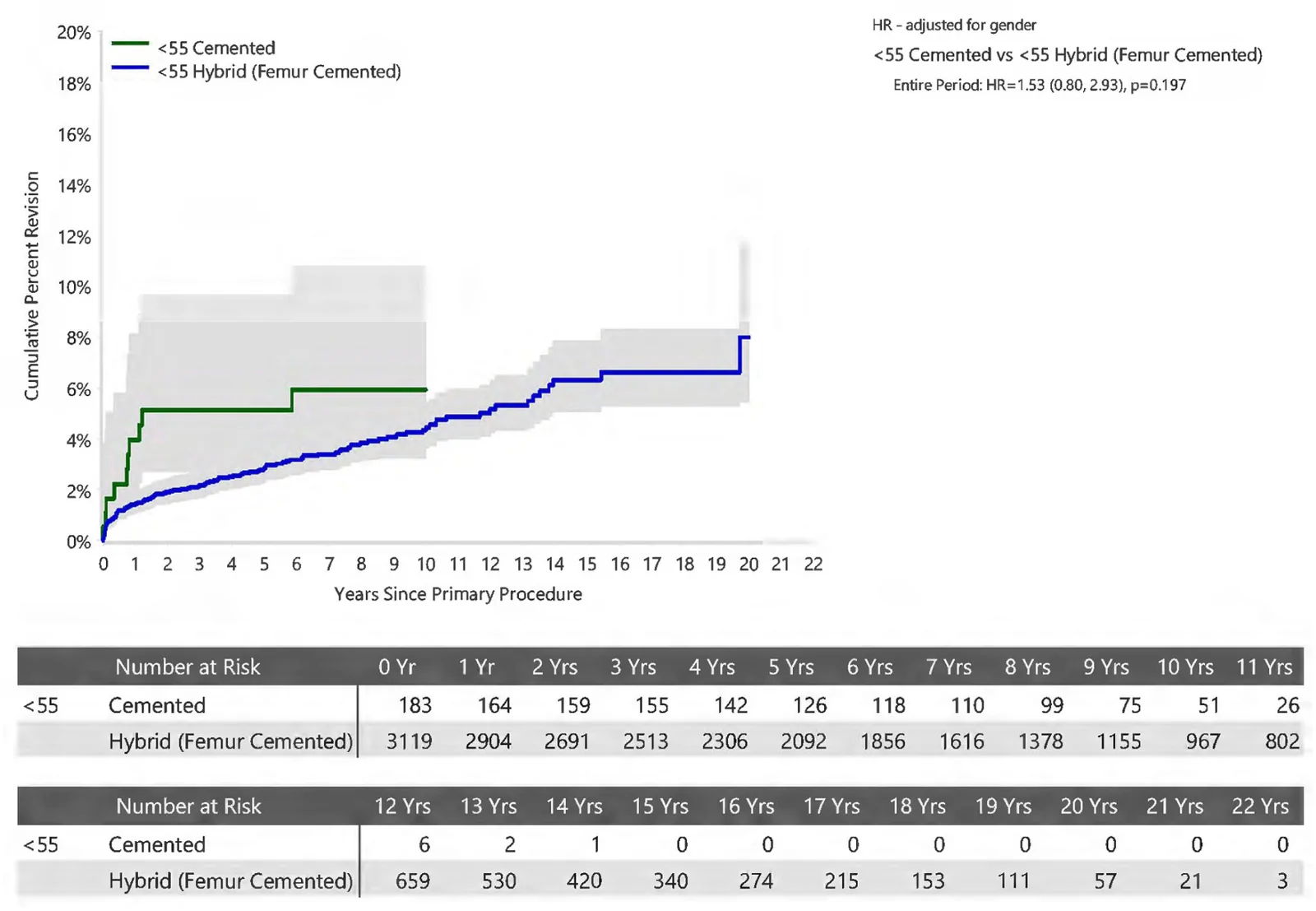
Cumulative Percent Revision of Primary Total Conventional Hip Arthroplasty in Patients Aged <55 years by Fixation (Primary Diagnosis Osteoarthritis).

**Figure 3.**
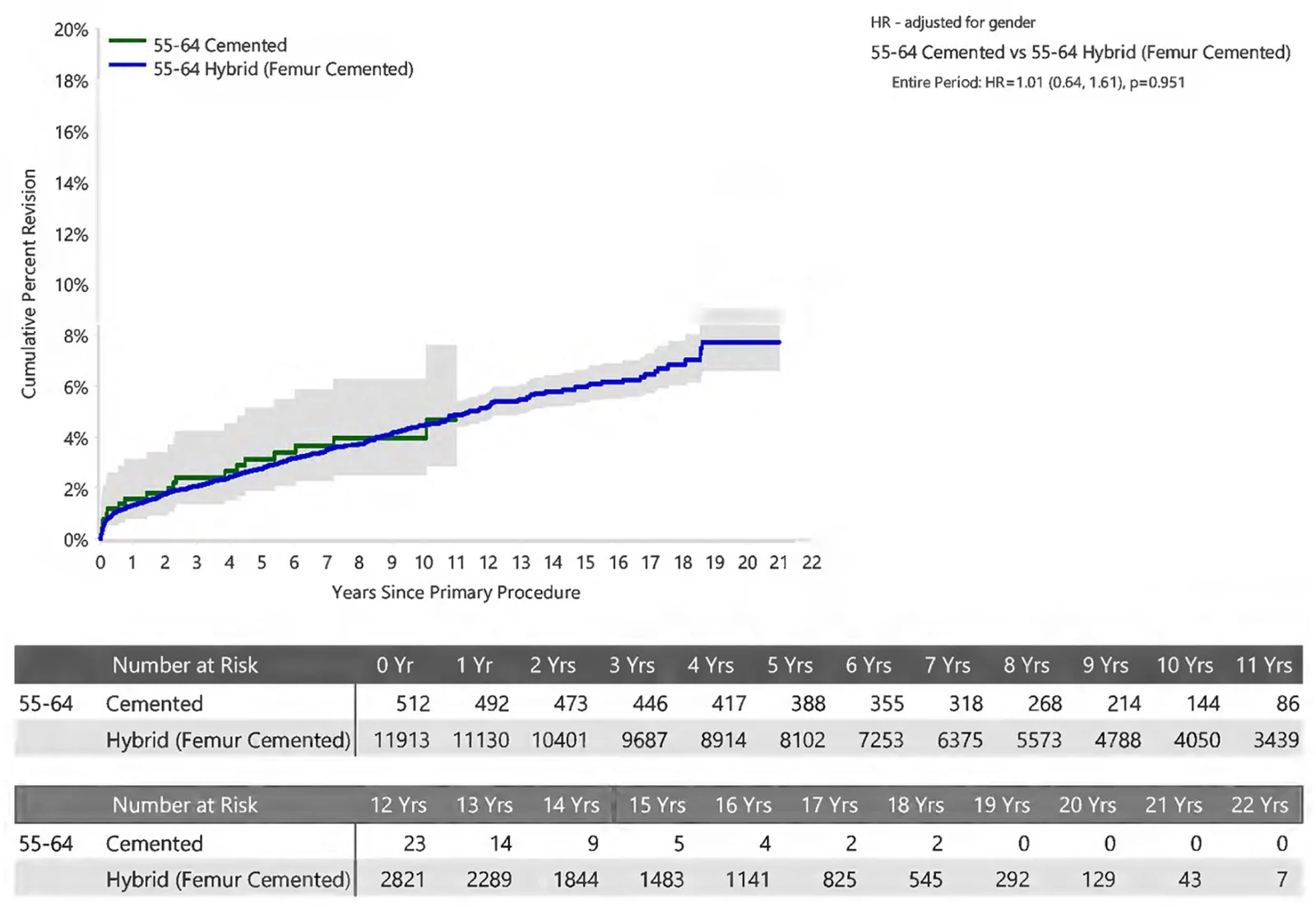
Cumulative Percent Revision of Primary Total Conventional Hip Arthroplasty in Patients Aged 55-64 years by Fixation (Primary Diagnosis Osteoarthritis).

**Figure 4.**
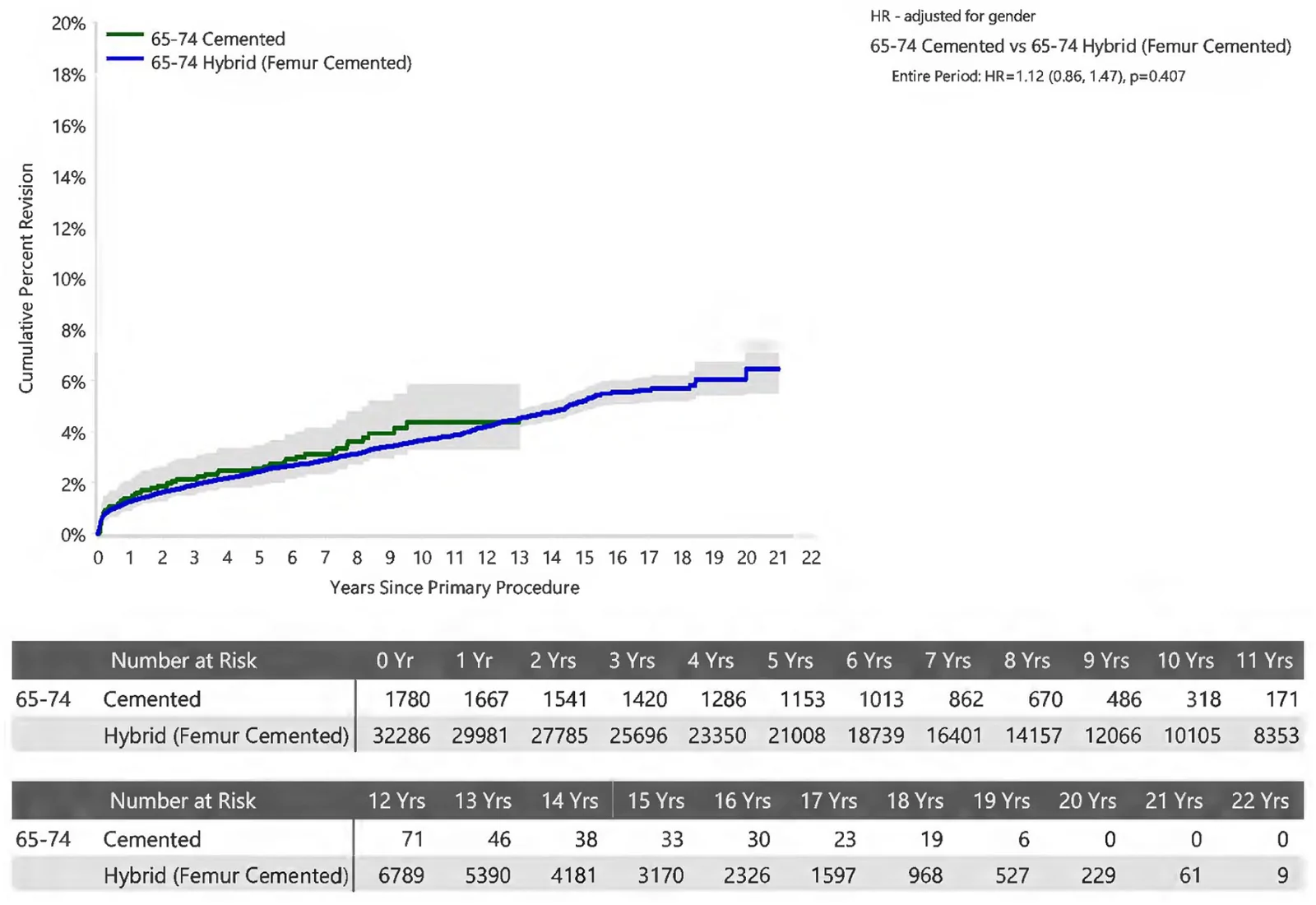
Cumulative Percent Revision of Primary Total Conventional Hip Arthroplasty in Patients Aged 65-74 years by Fixation (Primary Diagnosis Osteoarthritis).

**Figure 5.**
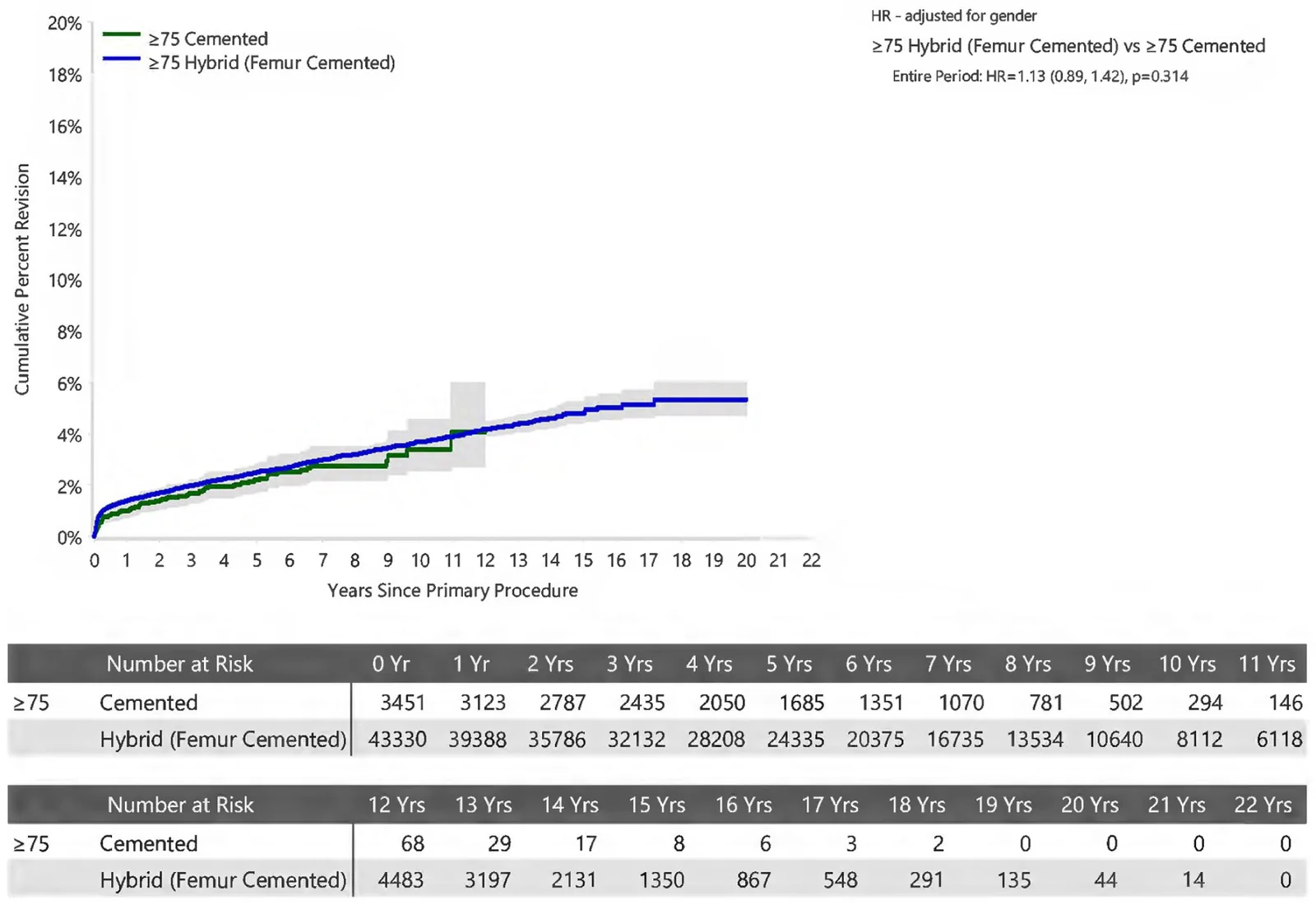
Cumulative Percent Revision of Primary Total Conventional Hip Arthroplasty in Patients Aged ⩾75 years by Fixation (Primary Diagnosis Osteoarthritis).

Stratification by patient age (Figures 2–5) revealed no statistically significant difference between acetabular fixation method.

**Figure 6.**
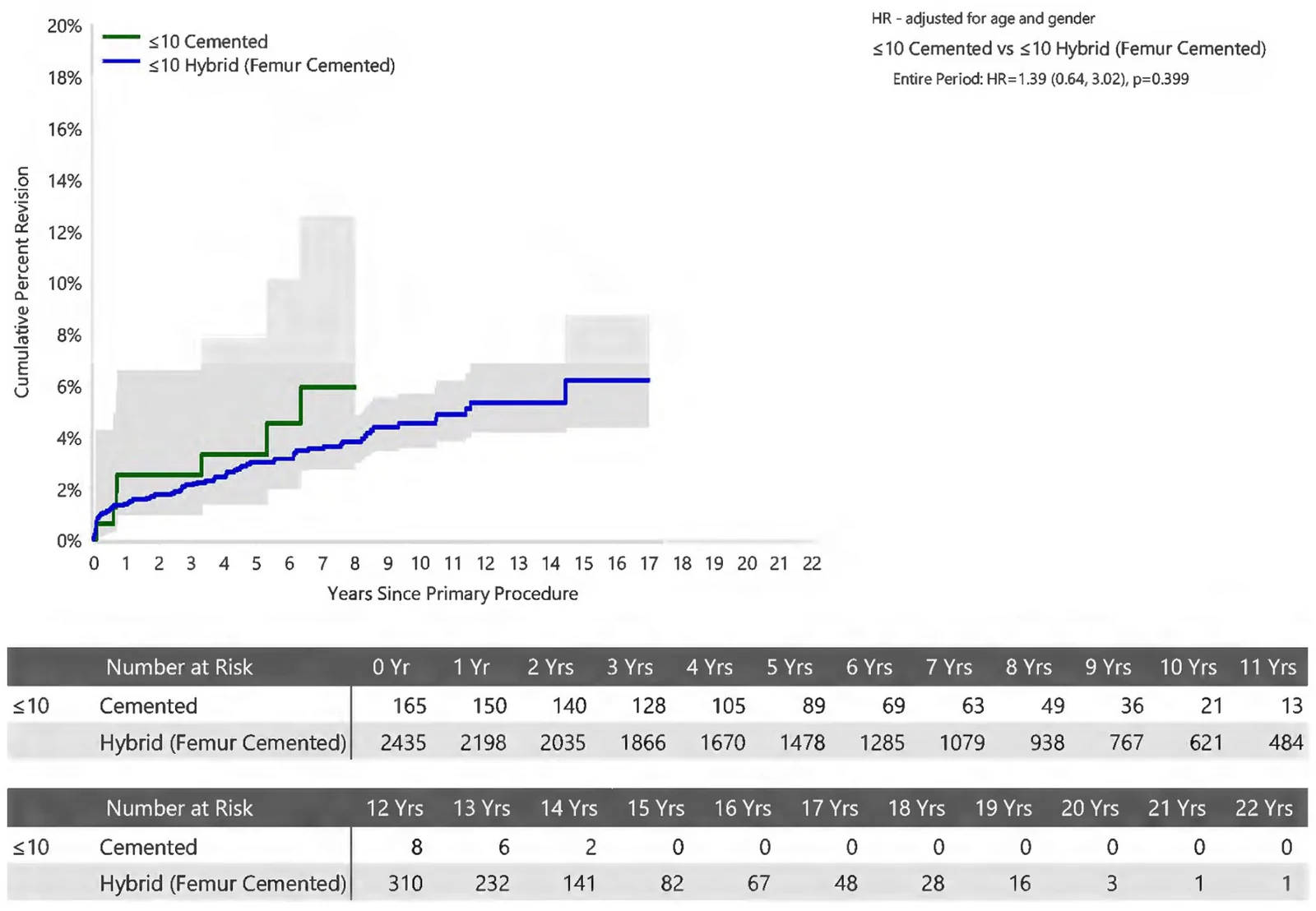
Cumulative Percent Revision of Primary Total Conventional Hip Arthroplasty for Surgeons performing ⩽10 procedures per year by Fixation (Primary Diagnosis Osteoarthritis)

**Figure 7.**
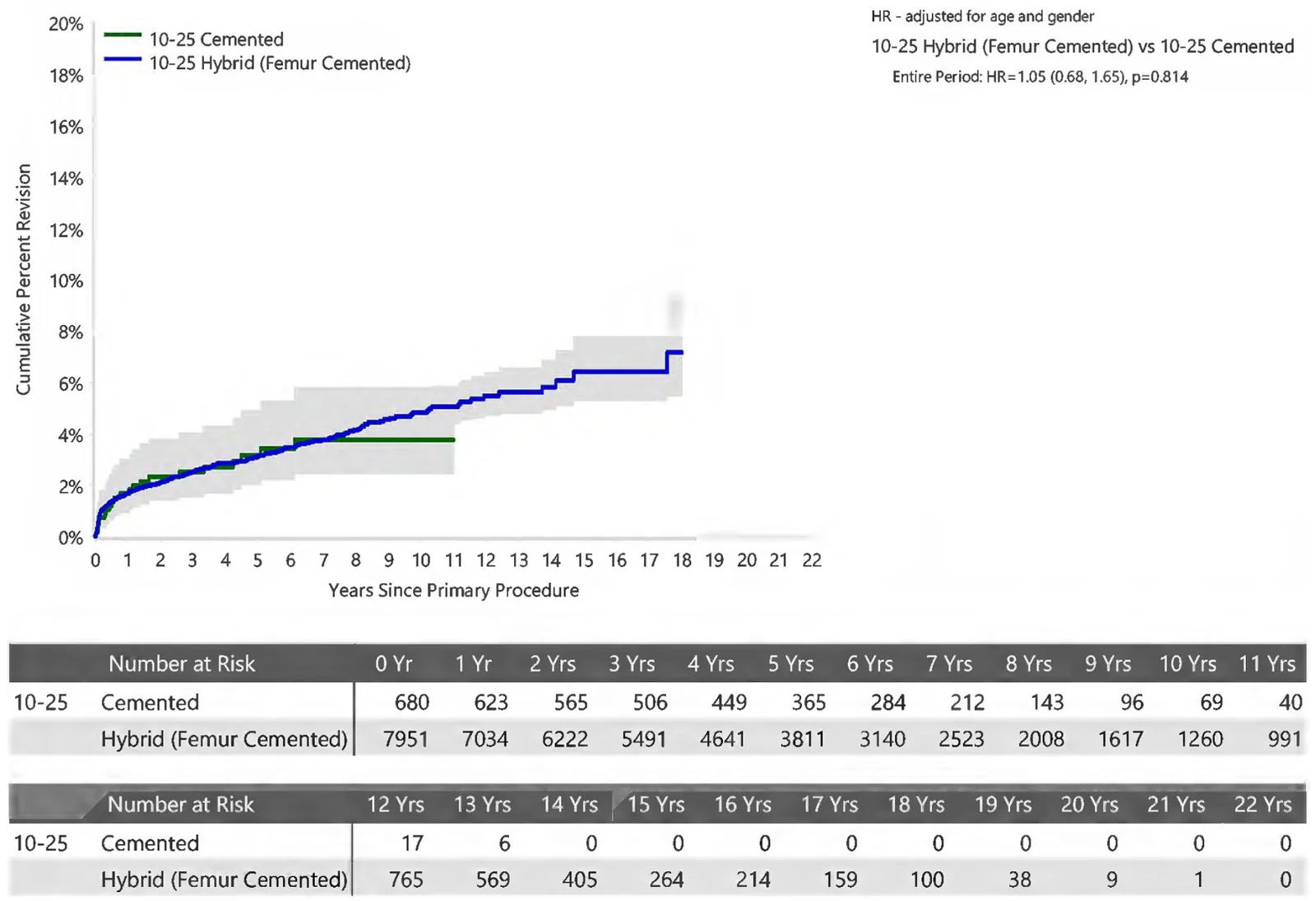
Cumulative Percent Revision of Primary Total Conventional Hip Arthroplasty for Surgeons performing 10-25 procedures per year by Fixation (Primary Diagnosis Osteoarthritis)

**Figure 8.**
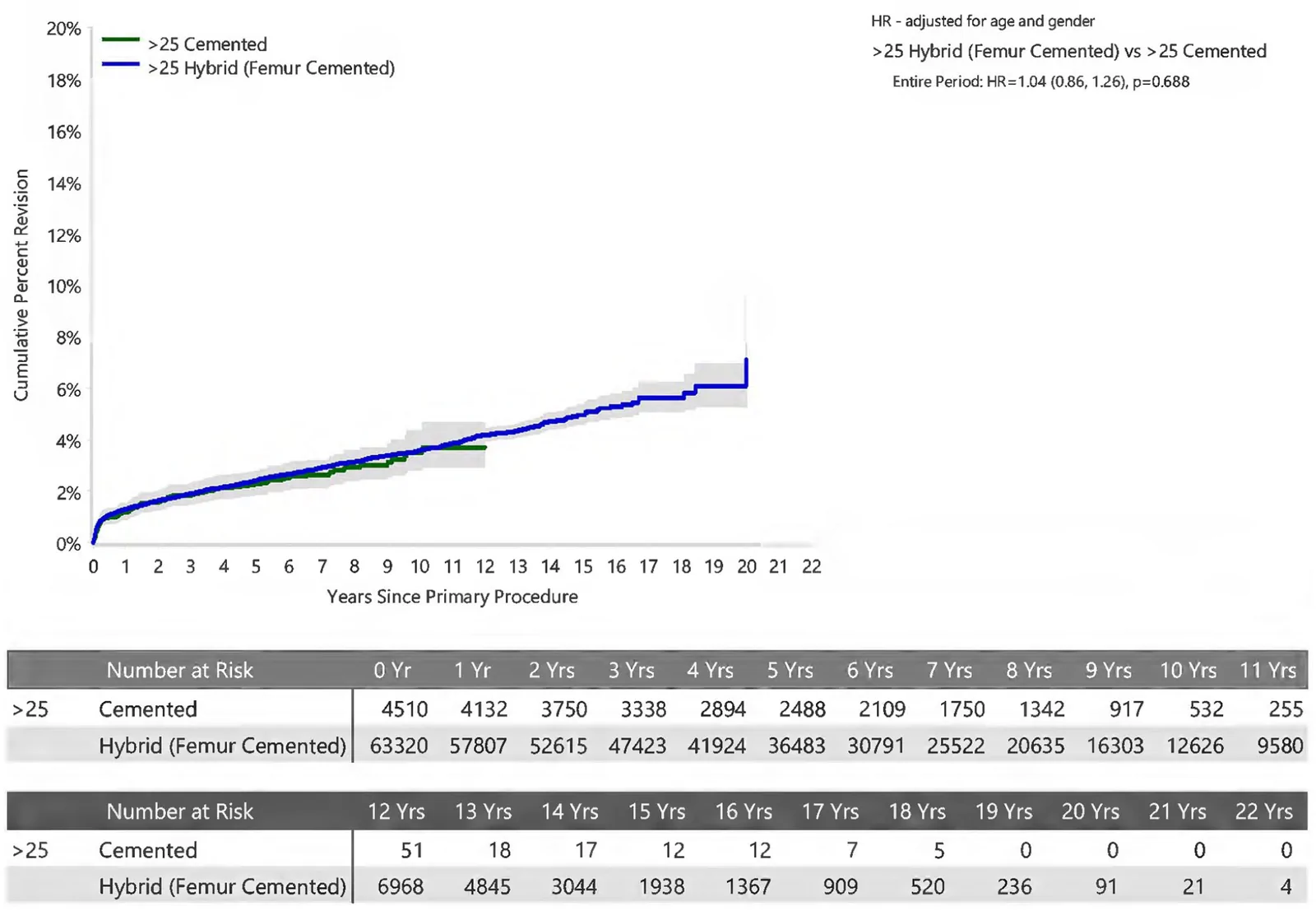
Cumulative Percent Revision of Primary Total Conventional Hip Arthroplasty for Surgeons performing >25 procedures per year by Fixation (Primary Diagnosis Osteoarthritis)

An analysis of surgeon volume demonstrated no statistically significant difference when comparing fixation methods within each volume stratification (Figures 6–8).

PROMs available for this cohort did not show any differences between groups on pre- and postoperative scores for EQ-5D, EQ-VAS, OHS and patient satisfaction scores. The detailed analyses are included in the Appendix.

## Discussion

This study demonstrates the equivalence of survivorship and functional outcomes in THA based on choice of acetabular component fixation. This reflects findings in the global body of literature.^2,18–20^ The endpoint of all-cause revision is a more inclusive and objective outcome measure than revision for aseptic loosening alone, as suggested by Toossi et al.^2^ The most common cause for revision in both groups was fracture. Revision for fracture was further classified based on componentry revised (Table 7). Of interest is that there were no isolated acetabular revisions for the cemented group. Furthermore, there has been increased recognition of periprosthetic fracture related to femoral stem design with increased rates of periprosthetic fracture with use of the CPT stem (Zimmer) resulting in recent product withdrawal.^21,22^ CPT stem use accounted for 3.7% of the cemented group and 13.0% of the hybrid group and demonstrated no difference in this analysis; this, however, represents a limitation of the study.^23^

Femoral head size has been implicated in revision risk profile with increased dislocation risk seen in head size 32 mm and smaller and increased aseptic loosening and fracture in head size >32 mm.^24^ The exclusion of non-XLPE limits the duration of follow-up in the cemented group, which is a potential limitation in the assessment of aseptic loosening. The increased proportion of 32-mm head use in the cemented group did not manifest an increase in revision for instability. Head size differences between groups showed no difference in all cause revision; however, a longer follow-up would be anticipated to assess the effect of larger head size on volumetric wear and aseptic loosening.

Technique specific implications associated with choice of acetabular fixation are revealed in the sub classification of revision for instability (Table 5). The inherent lack of modularity with cemented acetabular components, demonstrated by the increased acetabular revision rate of 63.9% (cf 27.7% for cementless) is juxtaposed with the modularity of cementless acetabular components affording the option liner exchange to vary offset, coverage, and constraint, conveyed by the increased minor revision rate for cementless acetabular components of 56.7% (cf 11.1% for cemented). Whilst there are technique specific nuances to be considered in the revision setting, the disparity in utilisation between methods of acetabular fixation cannot be explained or supported by a survivorship benefit.

The effect of surgeon volume and experience has been explored in the AOANJRR in its 2012 Annual Report.^25^ More recently, this has been reported by Malik et al.^26^ with an associated improvement in short- to mid-term survivorship as well as decreased perioperative complications and length of stay for higher volume surgeons. A proficiency threshold for cemented acetabular fixation of 10 cases per annum has been defined by the authors based on AOANJRR data as a level at which risk for early complication is mitigated.^27,28^ The data from this report demonstrates equivalence of fixation when stratified by volume of practice.

Age at time of surgery theoretically conveys an inversely proportional risk of revision based on the potential for increased activity levels over a longer period resulting in higher polyethylene component wear rates in younger patients.^29,30^ For a diagnosis of osteoarthritis there was no significant difference in all-cause revision rate when stratified by age groups <55, 55–64, 65–74 and ⩾75 years. This is supported by the results presented from the UK NJR showing no difference in revision rates for male patients <60 years regardless of prosthesis type.^20^ Female patients from the same cohort had a reported benefit with cementless acetabular prosthesis based on PROMs only.^20^ Surgeons may have chosen to use cementless fixation on more active patients. Age has been used as a surrogate for activity but we have not demonstrated any difference in revision rates when stratified for age. Cemented acetabular prostheses maintain equivalence across all age groups.

The motivation for surgeon choice of prosthesis is multifactorial and difficult to quantify.^31^ In attempting to quantify surgeon motivations and barriers to change, Vertullo et al.^31^ outline the myriad influences on a surgeon’s choice of TKR implant. Whilst these non-tangible factors are similarly likely to be contributory to prosthesis selection in THA, there is no comparative paper and the effect on outcome in this cohort is assumed to be limited. The AOANJRR reports 1519 different stem and acetabular combinations using modern prostheses (in current use) with 107 prosthesis combinations having >500 implantations in the 2023 Annual Report.^3^ With regard to cemented acetabular components, in 2022 there were 29 options with the 10 most commonly implanted accounting for 85.8% of total volume.^32^ By contrast there are 50 cementless acetabular component options in 2022 with the 10 most commonly implanted accounting for 88.2% of total volume.^3^ The differences between brands have been highlighted in the work of Jameson et al.^8^ The data from this report reflects equivalence of fixation methods in the context of a heterogenous profile of prosthesis choices. The decline in use of cemented cups may also be driven by perceived shorter operative time, although surgical time is not recorded in the AOANJRR so this cannot be supported.

There are inherent limitations with this registry study. Whilst use of a cemented acetabular prosthesis has additional utility in clinical settings of inflammatory arthropathy, malignancy, or infection, the authors felt that limiting the cohort to a diagnosis of osteoarthritis was necessary to minimise the confounding effect of including multiple diagnoses. There is limitation in the interpretation of peri-prosthetic fracture rate due to the AOANJRR data only capturing fractures requiring revision of componentry. The AOANJRR does not differentiate between acetabular and femoral fractures as a diagnosis for cause of revision which limits further subgroup analysis. Furthermore, the authors anticipate that the rate of open reduction and internal fixation (ORIF) for peri-prosthetic fracture would be unlikely to alter the reported results relating to choice of acetabular fixation. Observational studies such as these are vulnerable to being confounded by omitted variables. Demographic data pertaining to race, socioeconomic status, level of education, and perioperative pain levels were not available, though we believe that there was unlikely to be a difference between these groups. Radiological data was not available and is another potentially confounding factor when considering component malposition, instability, and wear related indications for revision as an endpoint. As the use of XLPE has been associated with minimal revisions for wear or osteolysis we believe this is unlikely to have been contributory.^33,34^

The relatively short mean follow-up of 7 years in this study is due to the inclusion of cases using XLPE only and is further reduced in the cemented group due to the later adoption of this technology in cemented cups. Longer term follow-up is required to monitor whether any differences, particularly in relation to late presenting reasons for revision such as aseptic loosening, occur.

## Conclusion

For patients undergoing a primary THA for osteoarthritis there is no difference in the rate of revision between cemented or cementless acetabular fixation when utilising a cemented polished stem with modern bearing surfaces. The authors advocate that the use of cemented acetabular fixation is a viable alternative and emphasise the need for continued teaching of the skills required to ensure the technique is not lost for future generations of orthopaedic surgeons.

## Appendix

**Table A1.** Preoperative EQ-5D-5L Mobility in Primary Total Conventional Hip Replacement by Fixation (Primary Diagnosis OA).

	Cemented		Hybrid (Femur Cemented)		TOTAL	
Preoperative EQ-5D-5L Mobility	N	Col%	N	Col%	N	Col%
No Problems	14	3.7	163	4.0	177	4.0
Slight Problems	45	11.7	530	13.1	575	13.0
Moderate Problems	133	34.7	1569	38.7	1702	38.3
Severe Problems	177	46.2	1698	41.9	1875	42.2
Unable to Do	14	3.7	97	2.4	111	2.5
**TOTAL**	**383**	**100.0**	**4057**	**100.0**	**4440**	**100.0**

**Table A2.** Postoperative EQ-5D-5L Mobility in Primary Total Conventional Hip Replacement by Fixation (Primary Diagnosis OA).

	Cemented		Hybrid (Femur Cemented)		TOTAL	
Postoperative EQ-5D-5L Mobility	N	Col%	N	Col%	N	Col%
No Problems	133	46.0	1466	54.8	1599	53.9
Slight Problems	88	30.4	679	25.4	767	25.9
Moderate Problems	45	15.6	382	14.3	427	14.4
Severe Problems	21	7.3	138	5.2	159	5.4
Unable to Do	2	0.7	11	0.4	13	0.4
**TOTAL**	**289**	**100.0**	**2676**	**100.0**	**2965**	**100.0**

**Table A3.** Preoperative EQ-5D-5L Pain/Discomfort in Primary Total Conventional Hip Replacement by Fixation (Primary Diagnosis OA).

	Cemented		Hybrid (Femur Cemented)		TOTAL	
Preoperative EQ-5D-5L Pain/Discomfort	N	Col%	N	Col%	N	Col%
No Pain	5	1.3	42	1.0	47	1.1
Slight Pain	42	11.0	448	11.1	490	11.1
Moderate Pain	147	38.5	1665	41.2	1812	40.9
Severe Pain	148	38.7	1500	37.1	1648	37.2
Extreme Pain	40	10.5	390	9.6	430	9.7
**TOTAL**	**382**	**100.0**	**4045**	**100.0**	**4427**	**100.0**

**Table A4.** Postoperative EQ-5D-5L Pain/Discomfort in Primary Total Conventional Hip Replacement by Fixation (Primary Diagnosis OA).

	Cemented		Hybrid (Femur Cemented)		TOTAL	
Postoperative EQ-5D-5L Pain/Discomfort	N	Col%	N	Col%	N	Col%
No Pain	110	38.3	1171	43.9	1281	43.4
Slight Pain	112	39.0	972	36.4	1084	36.7
Moderate Pain	48	16.7	396	14.8	444	15.0
Severe Pain	14	4.9	110	4.1	124	4.2
Extreme Pain	3	1.0	19	0.7	22	0.7
**TOTAL**	**287**	**100.0**	**2668**	**100.0**	**2955**	**100.0**

**Table A5.** Change in EQ-5D-5L Domain Score (N (%) in Primary Total Conventional Hip Replacement by Fixation (Primary Diagnosis OA).

	Worse		No Change		Better		TOTAL	
Outcome	Cemented	Hybrid	Cemented	Hybrid	Cemented	Hybrid	Cemented	Hybrid
Mobility	14 (5.2%)	96 (3.9%)	46 (17.2%)	350 (14.2%)	208 (77.6%)	2016 (81.9%)	268	2462
Personal Care	16 (6.0%)	134 (5.4%)	112 (41.8%)	994 (40.4%)	140 (52.2%)	1331 (54.1%)	268	2459
Usual Activities	19 (7.1%)	131 (5.3%)	55 (20.6%)	385 (15.7%)	193 (72.3%)	1938 (79.0%)	267	2454
Pain/Discomfort	12 (4.5%)	62 (2.5%)	36 (13.5%)	319 (13.0%)	218 (82.0%)	2071 (84.5%)	266	2452
Anxiety/Depression	22 (8.3%)	176 (7.2%)	137 (51.5%)	1238 (50.5%)	107 (40.2%)	1037 (42.3%)	266	2451

**Table A6.** Mean Pre-operative and Post-operative EQ VAS Health in Primary Total Conventional Hip Replacement by Fixation (Primary Diagnosis OA).

	Pre-operative		Post-operative	
Fixation	N	Mean ± SD	N	Mean ± SD
Cemented	377	64.16 ± 20.60	282	77.07 ± 17.69
Hybrid (Femur Cemented)	3992	64.61 ± 20.93	2650	79.85 ± 16.25
**TOTAL**	**4369**	**64.57 ± 20.90**	**2932**	**79.58 ± 16.41**

**Table A7.** Mean Postoperative OHS Health in Primary Total Conventional Hip Replacement by Fixation (Primary Diagnosis OA).

	Unadjusted		Complete Cases	
Variable	Estimate (95% CI)	p-value	Estimate (95% CI)*	p-value
Cemented	38.7 (37.8, 39.6)		40.3 (39.2, 41.4)	<.0001
Hybrid (Femur Cemented)	40.6 (40.3, 40.9)		41.3 (40.6, 41.9)	<.0001
Change: Cemented	19.5 (18.6, 20.4)		21.1 (20.0, 22.1)	
Change: Hybrid (Femur Cemented)	21.4 (21.1, 21.7)		22.0 (21.4, 22.7)	
**Difference: Cemented vs Hybrid (Femur Cemented)**	**−1.9 (−2.8, −0.9)**	**<.0001**	**−1.0 (−1.9, −0.0)**	**0.0436**

* Adjusted for age, gender, ASA score, BMI and pre-operative score.

**Table A8.** Primary Total Conventional Hip Replacement by Procedure Satisfaction and Fixation (Primary Diagnosis OA).

Procedure Satisfaction	Cemented N (%)	Hybrid N (%)	TOTAL N (%)
Very Satisfied	190 (66.7%)	1921 (72.5%)	2111 (72.0%)
Satisfied	61 (21.4%)	457 (17.3%)	518 (17.7%)
Neutral	14 (4.9%)	125 (4.7%)	139 (4.7%)
Dissatisfied	10 (3.5%)	55 (2.1%)	65 (2.2%)
Very Dissatisfied	10 (3.5%)	90 (3.4%)	100 (3.4%)
**TOTAL**	**285**	**2648**	**2933**

**Table A9.** Primary Total Conventional Hip Replacement by Patient-Reported Change and Fixation (Primary Diagnosis OA).

Patient-Reported Change	Cemented N (%)	Hybrid N (%)	Total N (%)
Much Better	250 (87.7%)	2435 (92.0%)	2685 (91.5%)
A Little Better	20 (7.0%)	132 (5.0%)	152 (5.2%)
About the Same	6 (2.1%)	42 (1.6%)	48 (1.6%)
A Little Worse	3 (1.1%)	23 (0.9%)	26 (0.9%)
Much Worse	6 (2.1%)	16 (0.6%)	22 (0.8%)
**TOTAL**	**285**	**2648**	**2933**

## References

[1] Jones LC Hungerford DS. Cement disease. Clin Orthop Relat Res 1987; 225: 192–206. 3315375

[2] Toossi N Adeli B Timperley AJ , et al. Acetabular components in total hip arthroplasty: is there evidence that cementless fixation is better? J Bone Joint Surg Am 2013; 95: 168–174. 23324965 10.2106/JBJS.K.01652

[3] Smith PN Gill DR McAuliffe MJ , et al. Hip, knee and shoulder arthroplasty: 2023 annual report. 2023. Adelaide, South Australia. Australian Orthopaedic Association National Joint Replacement Registry, AOA, Adelaide, SA, Australia.

[4] The NJR Centre. National Joint Registry for England, Wales, Northern Ireland and Isle of Man. 21st Annual Report 2024. Report no. 2054-183X. The NJR Centre, Hemel Hempstead, 2024.

[5] von Knoch F Malchau H . Why do we need a national joint replacement registry in the United States? Am J Orthop (Belle Mead NJ) 2009; 38: 500–503. 20011738

[6] Paxton E Cafri G Havelin L , et al. Risk of revision following total hip arthroplasty: metal-on-conventional polyethylene compared with metal-on-highly cross-linked polyethylene bearing surfaces: international results from six registries. J Bone Joint Surg Am 2014; 96(Suppl. 1): 19–24. 25520415 10.2106/JBJS.N.00460 PMC4271419

[7] Australian Orthopaedic Association National Joint Replacement Registry. Hip, knee & shoulder arthroplasty. 2016 Annual Report. Report no. 1445-3657, 2016. Adelaide: AOA.

[8] Jameson SS Mason JM Baker PN , et al. Factors influencing revision risk following 15 740 single-brand hybrid hip arthroplasties: a cohort study from a National Joint Registry. J Arthroplasty 2013; 28: 1152–1159. 23523210 10.1016/j.arth.2012.11.021

[9] Dumbleton JH Manley MT Sutton K. Ultra high molecular weight polyethylene. In: Ling RSM Lee AJC Gie GA , et al. (eds) The Exeter Hip – 40 years of innovation in total hip arthroplasty. Exeter: Exeter Hip Publishing, 2010, pp.57–62.

[10] Australian Orthopaedic Association National Joint Replacement Registry. Annual report. Report no. 1445-3657, 2014. Adelaide: AOA.

[11] de Steiger RN Muratoglu O Lorimer M , et al. Lower prosthesis-specific 10-year revision rate with crosslinked than with non-crosslinked polyethylene in primary total knee arthroplasty. Acta Orthop 2015; 86: 721–727. 26119884 10.3109/17453674.2015.1065046 PMC4750773

[12] Australian Orthopaedic Association National Joint Replacement Registry (AOANJRR). Hip, knee & shoulder arthroplasty: 2017 annual report. Report no. 1445-3657, 2017. Adelaide: AOA.

[13] Australian Orthopaedic Association National Joint Replacement Registry (AOANJRR). Hip, knee & shoulder arthroplasty: 2018 annual report. Report no. 1445-3657, 2018. Adelaide: AOA.

[14] de Steiger R Lorimer M Graves SE . Cross-linked polyethylene for total hip arthroplasty markedly reduces revision surgery at 16 years. J Bone Joint Surg Am 2018; 100: 1281–1288. 30063590 10.2106/JBJS.17.01221

[15] Zhou Y Wall CJ Stevens J , et al. Data resource profile: The Australian Orthopaedic Association National Joint Replacement Registry (AOANJRR). Int J Epidemiol 2025; 54: dyaf078. 10.1093/ije/dyaf078 PMC12199916 40524466

[16] Lewis PL Gill DR McAuliffe MJ , et al. Hip, knee and shoulder arthroplasty: 2024 annual report. 2024. Adelaide, South Australia. Australian Orthopaedic Association National Joint Replacement Registry, AOA, Adelaide, SA, Australia.

[17] Hoskins W van Bavel D Lorimer M , et al. Polished cemented femoral stems have a lower rate of revision than matt finished cemented stems in total hip arthroplasty: an analysis of 96,315 cemented femoral stems. J Arthroplasty 2018; 33: 1472–1476. 29310918 10.1016/j.arth.2017.12.002

[18] Bjørgul K Novicoff WM Andersen ST , et al. No differences in outcomes between cemented and uncemented acetabular components after 12-14 years: results from a randomized controlled trial comparing Duraloc with Charnley cups. J Orthop Traumatol 2010; 11: 37–45. 20198405 10.1007/s10195-010-0082-2 PMC2837808

[19] Hooper GJ Rothwell AG Stringer M , et al. Revision following cemented and uncemented primary total hip replacement: a seven-year analysis from the New Zealand Joint Registry. J Bone Joint Surg Br 2009; 91: 451–458. 19336803 10.1302/0301-620X.91B4.21363

[20] Jameson SS Mason J Baker P , et al. Have cementless and resurfacing components improved the medium-term results of hip replacement for patients under 60 years of age? Acta Orthop 2015; 86: 7–17. 25285617 10.3109/17453674.2014.972256 PMC4366667

[21] Windell L Kulkarni A Alabort E , et al. Biomechanical comparison of periprosthetic femoral fracture risk in three femoral components in a sawbone model. J Arthroplasty 2021; 36: 387–394. 32826144 10.1016/j.arth.2020.07.061

[22] Khan B McLauchlan GJ. Patient and technical contributors to periprosthetic fracture around the CPT stem. Hip Int 2023; 33: 1115–1121. 36703242 10.1177/11207000221140565

[23] Lewis PL Gill DR McAuliffe MJ , et al. Prostheses with higher than anticipated rates of revision. Hip, knee and shoulder arthroplasty: 2024 annual report. Adelaide, South Australia, 2024, pp.576–611. Australian Orthopaedic Association National Joint Replacement Registry, AOA, Adelaide, SA, Australia.

[24] Wallace DT Whitehouse SL Du P , et al. Does a 36-mm head increase cumulative revision rate in total hip arthroplasty when compared to a 32-mm head? A study from the Australian Orthopaedic Association National Joint Replacement Registry. J Arthroplasty. Epub ahead of print 18 August 2025. DOI: 10.1016/j.arth.2025.08.014. 40835012

[25] Australian Orthopaedic Association National Joint Replacement Registry. Annual report. Report no. 1445-3657, 2012. Australian Orthopaedic Association National Joint Replacement Registry, AOA, Adelaide, SA, Australia.

[26] Malik AT Jain N Scharschmidt TJ , et al. Does surgeon volume affect outcomes following primary total hip arthroplasty? A systematic review. J Arthroplasty 2018; 33: 3329–3342. 29921502 10.1016/j.arth.2018.05.040

[27] Van Bavel D Whitehouse SL Crawford RW , et al. Cemented vs cementless cups in total hip arthroplasty, a review of the AOANJRR. In: Australian Orthopaedic Association & New Zealand Orthopaedic Association annual scientific meeting. Cairns, 9–13 October, 2016.

[28] Hanly RJ Whitehouse SL Lorimer MF , et al. The outcome of cemented acetabular components in total hip arthroplasty for osteoarthritis defines a proficiency threshold: Results of 22,956 cases from the Australian Orthopaedic Association National Joint Replacement Registry. J Arthroplasty 2019; 34: 1711–1717. 31031154 10.1016/j.arth.2019.03.061

[29] Keeling P Howell JR Kassam AM , et al. Long-term survival of the cemented Exeter Universal stem in patients 50 years and younger: an update on 130 hips. J Arthroplasty 2020; 35: 1042–1047. 31882346 10.1016/j.arth.2019.11.009

[30] Migliorini F Maffulli N Pilone M , et al. Risk factors for liner wear and head migration in total hip arthroplasty: a systematic review. Sci Rep 2023; 13: 15612. 37730762 10.1038/s41598-023-42809-4 PMC10511625

[31] Vertullo CJ Grimbeek PM Graves SE , et al. Surgeon’s preference in total knee replacement: A quantitative examination of attributes, reasons for alteration, and barriers to change. J Arthroplasty 2017; 32: 2980–2989. 28552448 10.1016/j.arth.2017.04.035

[32] Australian Orthopaedic Association National Joint Replacement Registry (AOANJRR). Hip, knee & shoulder arthroplasty: 2020 annual report. Report no. 1445-3657, 2020. Adelaide: AOA.

[33] Deans CF Buckner BC Garvin KL. Wear, osteolysis, and aseptic loosening following total hip arthroplasty in young patients with highly cross-linked polyethylene: a review of studies with a follow-up of over 15 years. J Clin Med 2023; 12: 6615. 37892754 10.3390/jcm12206615 PMC10607435

[34] Jameson SS Baker PN Mason J , et al. The design of the acetabular component and size of the femoral head influence the risk of revision following 34 721 single-brand cemented hip replacements: a retrospective cohort study of medium-term data from a National Joint Registry. J Bone Joint Surg Br 2012; 94: 1611–1617. 23188900 10.1302/0301-620X.94B12.30040

